# Surgical management and outcome analysis of stage III pediatric empyema thoracis

**DOI:** 10.4103/0971-9261.69134

**Published:** 2010

**Authors:** Prema Menon, K. L. N. Rao, Meenu Singh, M. A. Venkatesh, R. P. Kanojia, R. Samujh, A. K. Saxena, Y. K. Batra

**Affiliations:** Department of Pediatric Surgery, Advanced Pediatric Center, Post Graduate Institute of Medical Education and Research, Chandigarh - 160 012, India; 1 Department of Pediatric Pulmonology, Advanced Pediatric Center, Post Graduate Institute of Medical Education and Research, Chandigarh - 160 012, India; 2 Department of Radio-Diagnosis, Advanced Pediatric Center, Post Graduate Institute of Medical Education and Research, Chandigarh - 160 012, India; 3 Department of Anesthesiology, Advanced Pediatric Center, Post Graduate Institute of Medical Education and Research, Chandigarh - 160 012, India

**Keywords:** Contrast-enhanced computed tomographic chest scan, decortication, empyema thoracis, pediatric

## Abstract

**Aim:**

Report of 125 pediatric patients of empyema thoracis treated by open decortication, highlighting the presentation, delay in referral, operative findings, the response to surgical intervention and follow-up.

**Materials and Methods:**

All the children who underwent open decortication for stage III empyema thoracis during the study period were included. Preoperative workup included hemogram, serum protein, chest radiographs and contrast-enhanced computed tomographic (CECT) scan of the chest.

**Results:**

One hundred and twenty-five patients (81 males, 44 females) (age 3 months–12 years, mean 4.9 years) were operated during a 4.5-year period. Among them, two children underwent bilateral thoracotomies. Also, 81.6% patients were referred 3 weeks after the onset of disease (mean duration 9 weeks). Intercostal chest drainage (ICD) had been inserted in (119) 95% cases. Thickened pleura, multiloculated pus and lung involvement were invariably seen on CECT scan. Bronchopleural fistula was present in 10 patients and empyema necessitatis in 2. Decortication, removal of necrotic tissue and closure of air leaks was performed in all the patients. Necrotizing pneumonia was seen in (35) 27.5% cases. Mean duration of postoperative ICD was 7 days. Follow-up ranged from 3 months to 4 years (mean 12 months). There was no mortality. Six patients had proven tuberculosis.

**Conclusions:**

The duration of the disease had a direct relationship with the thickness of the pleura and injury to the underlying lung. Delayed referral causes irreversible changes in the lung prolonging recovery. Only 18% presented within the early period of the disease. Meticulous open surgical debridement gives gratifying results. The status of the lung at the end of surgery is a major prognostic factor.

## INTRODUCTION

Empyema thoracis is a common disease. Children often present with persistent fever associated with cough, respiratory distress and occasionally chest pain. Most cases follow a bout of pneumonia which may have been particularly virulent in a child with poor nutritional status or immunity. We present our data from a tertiary care center, highlighting the presentation, delay in referral and the response to surgical intervention.

## MATERIALS AND METHODS

All consecutive patients during the period from August 2005 to December 2009, who underwent decortication were analyzed. Patients were investigated with complete hemogram, serum electrolytes and proteins, chest radiographs and a contrast-enhanced computed tomographic (CECT) scan of the chest before surgery. Indications for surgery were described by us in a previous report.[1] Both clinical symptoms and presence of debris/pus as well as degree of lung entrapment on CECT scan were taken into account. The advantages and morbidity after surgery were discussed with parents at length.

Surgery was done with a standard posterolateral thoracotomy through the 4^th^ or 5^th^ intercostal space. The thick parietal pleura appearing in the incision site was excised and all pus and debris in the pleural cavity removed. The visceral pleura were meticulously removed. The lung was allowed to expand, closing all air leaks detected with the help of manual ventilation. Necrotic lung tissue was removed and bronchopleural fistulas (BPFs) were closed. Chest tube drains were placed and the thoracotomy wound closed after giving an intercostal block.

The postoperative care included care of the chest drain. Chest radiographs were taken on day 1 postop, when the drain output was nil and after removal of the tube to rule out pneumothorax/collection. It was also done when there was no drainage but the patient was symptomatic. Ultrasound chest was found to be useful to differentiate between residual collection and consolidation. An extra chest drain had to be placed occasionally in some patients for drainage of a small pneumothorax or collection. Patients were started on steam inhalation and incentive spirometry as early as possible along with nursing in an upright position. Adequate pain relief and antibiotics were given intravenously till good oral intake resumed. The antibiotics were changed depending on the culture report of the pus/debris sent at the time of surgery. Patients were followed up at 3 months and 1 year or more frequently in the presence of symptoms.

## RESULTS

One hundred and twenty-five patients were treated. There were 44 girls (M:F = 1.84:1). Age ranged from 3 months to 12 years in boys (mean 5 years) and from 7 months to 11 years in girls (mean 4 years). Decortication was performed on the right side in 63 patients, on the left in 60, while 2 patients underwent bilateral thoracotomies. More boys presented below 1 year with an incidence of 17.3%, with only 6.9% girls presenting in infancy. More children presented in the 1–5 year age group compared to 5-12 years in both the sexes. There was a peak incidence during summers and rainy season (43%) compared to rest of the year.

History of fever and cough was the commonest feature [Table 1]. Respiratory distress often appeared to be the triggering factor for referral. Also, 31.5% were below the 10^th^ centile and 57.1% children were in the 10th-50th centile for weight as per the percentile chart for Indian children. Only 11.4% were in the 50th-90th centile. This indicated the severe malnourished status in these patients. Serum albumin ranged from 1.1 to 3 g% with an average of 1.7 g%. Pallor was common with mean Hb 8.7 g% (5.5–10.9 g%).

**Table 1:** Presenting features of empyema thoracis

Symptoms	No. of patients(%)
Fever	119 (95)
Cough	100 (80)
Respiratory distress	70 (56)
Chest pain	20 (16)
Abdominal pain	1
Hemoptysis	1
Loss of weight, appetite	11
Vomiting	3
Anasarca	4
Septic shock	2
Pericardial effusion	1
Empyema necessitatis	2

Nil to 50% lung volume was seen in the lung window of the CECT scan in most patients. There was variable amount of pus, air and debris between the thickened parietal and visceral pleura. In majority of them, multiloculations were present. In 57% cases, there was encasement of the entire lung with collapse consolidation, whereas in the remaining, part of the lung was spared.

Intercostal chest drain had been inserted before referral in 119 patients (95%). In 11 patients, it had been inserted twice. Some patients had been tapped several times at different centers based on opacity on chest radiograph alone. The drain had been kept for a period with a mean of 3 weeks. Unsuccessful use of streptokinase injection was also noted in some hospitalized patients.

While 64% preoperative pus cultures were sterile, 36% were positive and mostly grew *Staphylococcus, Pseudomonas*, and occasionally, *Acinetobacter*. The most commonly isolated organism was *Pseudomonas aeruginosa*. In the postoperative cultures, a few patients grew α and β hemolytic *Streptococcus, Klebsiella* and *Pseudomonas*. In the majority, it was reported as sterile. Before referral, nearly all the patients had been administered multiple antibiotics and 11 patients had been started on anti-tuberculous treatment empirically due to persistent symptoms. Six patients in the series were proven to have tuberculosis on histopathological examination of pleural/lung biopsy. The tuberculosis patients had similar favorable outcome except for 2 who had prolonged inter costal tube drainage (ICTD) drainage. One out of these required redo thoracotomy due to collection. A few non-tubercular and nonbacterial etiologies seen were mucormycosis (1), lymphoid pneumonia (1) and trauma (1).

Of the 125 patients, only 23 (18.4%) presented to us before 3 weeks of onset of illness. Eighty-seven patients (69.6%) were referred 3 weeks–3 months after the start of symptoms. Another 11 patients presented between 3 months and 1 year, and in the remaining (4 patients) the symptoms were present for 1–2 years. A comparison of patients presenting before and after the duration of 3 weeks was done [Table 2]. It can be observed that 81.6% of the cases were delayed referrals. As a consequence of delay, they had a higher incidence of lung necrosis and required lobectomies. The recovery time in these patients was higher (maximum 1 month) as compared to early patients (maximum 8 days). The ICTD drainage was also prolonged. Eight patients in this series were on ventilator at the time of referral [Table 3]. All patients after surgery were weaned off the ventilator and recovered with early removal of intercostal drains and good lung expansion.

**Table 2:** Comparison of patients presenting before and after 3 weeks of onset of empyema

Patient details	<3 weeks	>3 weeks
No. of patients	23 (18.4%)	102 (81.6%)
Bilateral	1	1
Mediastinal shift, deformed chest	1	1
Bilateral	0	7
BPF	2	8
Empyema necessitates	1	1
Pre-op ventilatory support	3	5
Incorrect pre-op diagnosis	CLE (1) Pulmonary mass(1)	CLE (1) CCAM (1) Lung cyst (2)
Operative findings		
Collection		
Air	(2)	(10) (associated adhesions in 3)
Pus	(8)	(55)
Debris, pus	(10)	(9)
Debris	(4)	(29)
Pleura		
Thin	4	1
Thick	20	84
Very thick (>1 cm)	0	18
Status of lung		
Consolidation	(6)	(15)
Necrosis	(7)	(28)
Poor compliance/fibrosis	(0)	(16)
Good lung expansion	(11)	(44)
Segmental lung resection	0	21
Lobectomy	1	5
Pneumonectomy	0	1
Time to discharge from hospital	3–8 days	5 days–1 month
Prolonged post-op ICTD (>1 month)	3	12
Wound infection	1	4

CLE (1): congenital lobar emphysema, CCAM (1): congenital cystic adenomatoid malformation

**Table 3:** Details of patients on preoperative ventilatory support

Age/sex	Affected side	Operative findings	Procedure
3 months/M	Bilateral empyema	Rt thoracotomy BPF, chest tube in middle lobe, very thick pleura	Rt middle lobectomy for necrotizing pneumonia
		Lt resolved with ICTD	
4 months/M	Bilateral empyema	Rt thoracotomy whole lung collapsed, very thick pleura	Rt decortication with good expansion of lung
		Lt resolved with ICTD	
1.5 years/F	Bilateral empyema	Bilateral thoracotomy	Removal of pus and debris
		Lt lung completely collapsed	Decortication with good expansion of lung
		Rt lower lobe collapsed	
3 years/M	Suspected pulmonary mass	Rt thoracotomy, upper lobe consolidation with no aeration; chest tube within necrotic lower lobe	Decortication, removal of necrotic tissue and closure of air leaks
	Rt empyema		
3 years/F	Purulent pericardial effusionpericadiotomy	Left thoracotomy, BPF	Decortication, pus and debris removal, closure of fistula
	Lt empyema with BPF	Thick pus and debris, Thick pleura	
8 years /M	Septic shock	Right thoracotomy	Decortication, pus and debris removal, closure of fistula
	Rt empyema with BPF	Collapsed upper and middle lobes	
		Upper lobe BPF	
8.5 years/F	Septic shock (staphylococcal)	Right thoracotomy	Decortication, good expansion of the upper and lower lobe with poor expansion of the middle lobe
	Rt empyema	Collapse UL, LL consolidation ML	
		Thick pleura, 50 ml debris	
11 years/F	Septic shock (post-traumatic)	Left thoracotomy	Decortication with complete expansion of collapsed lung
	Persistent Lt hydropneumothorax Only 10% lung volume on CT scan	Multiloculated pus, debris and air (>200 ml), thick pleural peel	

There was evidence of BPF in 10 patients, with leakage of large amount of air in the chest drain. Amongst them two patients were on ventilatory support at the time of referral, with one arriving in shock to the hospital. The previously inserted intercostal drain was seen within the lung parenchyma in four patients. Three patients underwent lobectomy of necrotic lung. In the rest, the air leaks were closed with debridement of local necrotic tissue. Only one patient had prolonged ICT drainage of more than 1 month after surgery. Rest of them recovered quickly. Two patients were referred with a diagnosis of congenital lobar emphysema and in fact had a loculated pneumothorax. Two patients presented with empyema necessitans with associated necrotizing involvement of thoracic muscles.

The main lung changes seen were necrotizing pneumonitis, consolidation and poor compliance with poor expansion [Table 4]. Complete expansion of the entire lung after decortication was seen in only 43.4% cases. These patients had a faster time to recovery and removal of chest drain. Chest drains placed at the time of surgery could be removed within 3-14 days in the majority of patients. Twelve patients were discharged home with the drain *in situ* due to financial constraints and/or prolonged drainage. In the presence of cavitary necrosis, patients who underwent segmentectomy or lobectomy had faster recovery than those who underwent only debridement. Associated lung hepatisation prolonged recovery.

**Table 4:** Comparison of lung changes and its effect on morbidity

	Necrotizing pneumonitis	Consolidation + poor expansion of	Compressed lung fully
		
		Lung/fibrosis	Expanded after decortication
No.	35 (27.5%)	37 (29.1%)	55 (43.4%)
Duration of illness	2 weeks–7 months	2 weeks–2 years	2 weeks–3 months
Median	2.5 months	1.5 months	1 month
Duration of pre-operative ICTD	8 days–6 weeks	7 days–4 weeks	4 days–8 weeks
BPF	10	Nil	Nil
Thoracotomy collection	Pus and air (16)	Pus and air (8)	Pus, air (25)
	Pus, debris, air (14)	Pus, air, debris (21)	Debris, pus, air (25)
	Air (5)	Air (8)	Air/nil (5)
Pre-op ventilatory support	4	1	3
Lung resection	25	3	Nil
Post-op ICTD	4 days–2 years Median (2 weeks)	5 days–9 months Median (8 days)	3–11 days Median (5 days)
Post-op cough/fever	3	3	2
1 year follow-up available and doing well	29	31	46
Specific etiology		Mucormycosis 1, TB 4	TB 2

Follow-up ranged from 3 months to 4 years (mean = one year). No procedure-related or delayed deaths were seen. Weight gain was seen in nearly all patients, 2 months after surgery. Eight patients complained of occasional fever and cough. Five patients had wound infection. One patient with tuberculosis underwent thoracotomy thrice for persistent drainage of air and pus. There was loss of lung tissue and poor expansion of the remainder. Two patients had residual pneumothorax/recurrent collection for which chest tube had to be reinserted for a few days.

## DISCUSSION

Empyema thoracis is seen all over the world with an apparent increase in its incidence in the West.[2,3] In countries like Australia where a national database for disease statistics exists, the incidence of empyema was found to be 7.35 per millionwith an increased incidence in 1-4 year age group. Additionally, the overall percentage of empyema as a proportion of pneumonia increased from 0.27 to 0.70% (*P* < 0.05) (3). In our own experience, over the past few years, there appears to be an increasing awareness amongst pediatrician colleagues over the requirement and benefits of decortication, which has led to a higher referral rate.

The primary aim in the early stages of empyema is to drain the pus completely. Controversies abound on the correct mode of management. Even developed countries which like to follow protocols do not have any clear-cut guidelines[3] Recent studies have even raised the issue whether patients with empyema should now bypass medical thoracostomy totally and proceed directly to video-assisted thoracoscopic surgery (VATS).[4] The pleural collection is often solid debris unlikely to come out through a catheter. The role of VATS in stage III empyema is however very much debatable.[5]

Patients have usually been seen by local physicians in the presence of fever and cough and put on several courses of broad-spectrum antibiotics. They tend to come to a tertiary center with the onset of respiratory distress. Chronic cough should not be assumed to be due to tuberculosis. Eleven patients had been started on anti-tuberculous treatment empirically due to persistent symptoms. Only two of them and another four patients in the entire series were eventually proven to have tuberculosis.

In this series, intercostal chest drain had been inserted in 119 patients (95%). This had been kept for a median of 3 weeks. Several patients had more than one intercostal tube inserted blindly based on a chest radiograph. Several others were repeatedly poked with needles as the earlier tap was dry. One has to remember that not only multiloculation but also thick debris resembling cheese will give a dry yield. Sometimes, after an initial drainage of pus, the only content is air, which remains because the lung is unable to expand, caged as it is by the thick unyielding visceral pleura. It is worth repeating here that the true nature of the pleural collection and the status of the lung can be determined only by a CECT scan.[1,6] Most centers the world over tend to manage complications of pneumonia with chest radiographs alone or at the most with an ultrasonography.[1] The world is slowly waking up to the importance of other modalities of imaging for timely detection and intervention of pleural space infections.[7]

Since we are in a tertiary/quaternary referral unit, patients invariably present at a late stage of the disease. Only 18.4% patients in our series gave a history of less than 3 weeks. Even in this group, thin pleura was seen at thoracotomy only in four patients. In most cases, the pleura had become a hard, leathery peel densely adherent to the underlying lung. However, we advice surgery only based on the clinical features (persistent high grade fever, cough, respiratory distress, irritability, poor appetite, etc.) along with corroboration by CECT scan and not on the duration of the disease.[1] Some patients who have been referred 2-3 weeks after the onset of disease have been continued on medical management when they are symptomatically improving, i.e. regression of fever, reducing contents in a chest drain in the presence of an expanding lung on chest radiograph, etc.

We found an increased incidence between May to August every year. A study from Taiwan also showed that the incidence of both pneumonia and empyema was highest each spring.[8] Another study from Canada showed that while admissions occurred throughout the year, they peaked between November and April.[9]

Most patients had received one or more courses of antibiotics, with 11 patients having been started on antituberculous treatment empirically. Of these, only two patients were proven to have tuberculosis on histopathological specimen of pleural/lung biopsy. There was evidence of BPF in 10 patients, with leakage of large amount of air in the chest drain, 2 of them arriving in hospital in a state of shock. At thoracotomy, the previously placed intercostal drain was found within the lung tissue in four patients. While a few underwent lobectomy of the necrotic lung, in the others the fistula could be closed after local debridement.

Malnutrition was evident in most patients at the time of admission, with 88% being below the 50th percentile for weight. Serum albumin averaged 1.7 g%. This may be secondary to the disease process or the disease itself may have manifested in a child with poor nutritional status. The latter appears more valid as only 13% patients who presented before 3 weeks of illness had a weight above the 50th centile. A higher complication rate has been observed in pediatric empyema patients with lower hematocrit on admission.[10] Pallor at presentation was common with preoperative Hb averaging 8.7 g% in our series. This average was much lower in children presenting after 3 weeks (7.67 g%) compared to the children who presented before 3 weeks. We usually transfuse whole blood preoperatively when the Hb is below 8-9 g%.

Three significant changes which affected morbidity were noted in the lung at thoracotomy: (a) consolidation, (b) cavitary necrosis and (c) poor compliance/fibrosis with poor expansion on Ambu bag ventilation. All these prolonged the recovery and removal of intercostal drain [Table 4]. Because of persistent pneumonitic changes, postoperative fever and cough can occur, and occasionally reinsertion of chest tube for a few days may be necessary. In a recent study from Brazil, 52 children who had undergone thoracoscopy for empyema were retrospectively studied, comparing patients with and without necrotizing pneumonia.[11] The incidence of thoracic drainage prior to thoracoscopy (79–36%), pneumothorax (46–32%) and BPF (67–18%) was far lesser in the second group. The hospital stay was also lesser in this group. In our series, BPFs were associated with necrotizing pneumonia or to a wrongly inserted chest drain. In our series, surgeons with varying experience including trainees have operated. The key to early recovery and early removal of the chest tube appears to be complete removal of necrotic lung tissue. This often entails lobectomy which some surgeons are reluctant to do leading to delayed recovery. Patients with persistent consolidation are more difficult to treat as excision is not the answer here.

One has to wait patiently for the lung to heal. Some patients had to be discharged home on open tube thoracostomy. There were only two patients with nearly complete involvement of the entire lung, one due to chronic compression by the thick pleura and the other with underlying tuberculosis. However, no patient underwent obliteration of pleural space by muscle/omental flaps or thoracoplasty.

To the best of our knowledge, this is the largest series of open thoracotomies for chronic pediatric empyema thoracis to be reported in the literature from a single institution.[12] Our study has shown that most cases of empyema are secondary to pneumonia. Tuberculosis is a rare causative agent accounting for only 4.7% cases. Delayed referral increases morbidity by not only increasing parenchymal involvement but also causing a state of malnutrition. The effect on the lung by the pneumonitic process and the compression by the thickened pleura which has stopped physiologically functioning has a variable effect. Apart from necrosis and consolidation which may be similar in patients presenting before and after 3 weeks, the incidence of reduced compliance of the lung and loss of lung tissue in chronic cases has a major effect on postoperative recovery. One should strive to correctly treat these patients at least when they are in stage II of their disease.
